# Complete genome sequence of *Cutibacterium acnes* type II CCSM0331, isolated from a healthy woman’s skin

**DOI:** 10.1128/mra.00108-23

**Published:** 2023-12-01

**Authors:** Laiji Ma, Yan Li, Yujie Niu, Suzhen Yang, Li Shao

**Affiliations:** 1 School of Perfume and Aroma Technology, Shanghai Institute of Technology, Shanghai, China; 2 R&D Innovation Center, Shandong Freda Biotech Co., Ltd, Jinan, Shandong, China; University of Maryland School of Medicine, Baltimore, Maryland, USA

**Keywords:** *Cutibacterium acnes*, complete genome sequence

## Abstract

The complete genome sequence of *Cutibacterium acnes* Type II strain CCSM0331, which was isolated from the healthy facial skin, is reported. The assembled 2.5-Mbp genome comprised a single circular chromosome. These data will provide valuable information on the beneficial role of *C. acnes* as a skin commensal bacteria.

## ANNOUNCEMENT

*Cutibacterium acne*s is usually associated with the development of acne vulgaris, commonly called acne (1 – 3). Recent research has shown that *C. acnes* is known as a skin-sentinel bacterium and plays a crucial role in maintaining skin homeostasis (4 – 8).

In 2021, strain CCSM0331 was isolated from the skin microbial sample collected from the cheek of a healthy young woman in Shanghai, China (approval no. 2021-KY-15). The sample was mixed, serially diluted with sterile water, and then cultured on tryptic soy agar (TSA) plate containing 5% defibrated sheep blood. After anaerobic incubation at 37°C for 3–4 days, small and white colonies were picked out and then re-steaked at least three times on TSA plate to obtain pure cultures. Strain CCSM0331 was identified as *C. acne*s (GenBank accession: OQ874071) using the universal primers 27F and 1492R by 16S rRNA gene sequencing (9).

The genomic DNA of CCSM0331 was extracted from 72 h anaerobically cultured in tryptic soy broth at 37°C using Wizard Genomic DNA Purification Kit (Promega). Genomic DNA was sequenced using a combination of PacBio RS II Single Molecule Real Time (SMRT) and Illumina sequencing platforms. For Illumina sequencing, DNA samples (at least 1 µg) were sheared into ~400 bp fragment using a Covaris M220 Focused Acoustic Shearer, and a library was constructed using the NEXTflex Rapid DNA-Seq Kit and then were used for paired-end Illumina sequencing (2 × 150 bp) on an Illumina Novaseq 6000, yielding 4,299,055 reads, and Q20 value of 96.7%. The sequencing reads were subjected to quality-filtered using fastp v.0.23.0 (10), yielding 4,186,122 reads, and Q20 value of 97.37%. For Pacific Biosciences sequencing, DNA samples (at least 10 µg) were processed into ~10 kb segments using a Covaris g-TUBE (Covaris, MA), and DNA fragments were then purified, end-repaired, and ligated with SMRTbell sequencing adapters (Pacific Biosciences, CA). The resulting sequencing library was purified three times using 0.45 × volumes of Agencourt AMPure XP beads (Beckman Coulter Genomics, MA). Next, an ~10 kb insert library was prepared and sequenced on one SMRT cell. The raw sequencing reads were processed using SMRT Analysis v.2.3.0, yielding 256,895 reads, an average read length of 11,656.58 bases, and *N*_50_ value is 12,540 bp.

The Illumina and PacBio read data were assembled *de novo* using Unicycler v.0.4.8 (11) to yield structurally consistent assembled sequences, which were then iteratively polished with Pilon v.1.22 (12) to generate a single circular chromosome of 2,496,923 bp with a GC content of 60.02%, and coverage depth reaches 1,199.28-fold. Rotation and circularity were confirmed via Unicycler v.0.4.8. The coding sequences of chromosome, tRNA, and rRNA were annotated by NCBI Prokaryotic Genome Annotation Pipeline (https://www.ncbi.nlm.nih.gov/genome/annotation_prok/), v.6.5. A total of 2,352 coding sequences (CDS), 9 rRNAs, and 45 tRNAs were predicted. Default parameters were used except where otherwise noted. These data will provide valuable information to research the functions of this strain.

## Data Availability

The complete nucleotide sequence of *C. acnes* CCSM0331 has been deposited in GenBank under accession number CP114143.1, BioProject PRJNA895196, BioSample SAMN31566135, and Sequence Read Archive (SRA) accession numbers SRP421094 and SRR23345837.
